# Patients’ trust in healthcare providers and associated factors among hospitalized patients in Awi Zone Public Hospitals, Northwest Ethiopia

**DOI:** 10.1371/journal.pone.0350498

**Published:** 2026-06-29

**Authors:** Baymot Bitew Bekele, Kelemework Gashinet Ferede, Abebaw Bires Adal, Addisu Gasheneit Ferede, Atsedemariam Andualem, Desalegn Yeshalem Mitiku, Aragaw Egziabherfenta Tadele, Asrat Yazew, Birhaneslasie Gebeyehu Yazew

**Affiliations:** 1 Department of Nursing, College of Medicine and Health Sciences, Injibara University, Injibara Ethiopia; 2 Department of Epidemiology and Biostatistics, College of Medicine and Health Sciences, Bahir Dar University, Bahir Dar, Ethiopia; 3 Institute of Public Health, College of Medicine and Health Sciences, University of Gondar, Gondar, Ethiopia; PLOS: Public Library of Science, UNITED STATES OF AMERICA

## Abstract

**Background:**

Trust in healthcare providers (HCPs) is crucial for increasing health-seeking behavior, strengthening patient-provider relationships, and improving clinical outcomes. While vital, research on patient trust in HCPs remains limited in the Ethiopian context. This study assessed the level of trust and its associated factors among hospitalized patient in Awi Zone public hospitals, Northwest Ethiopia.

**Methods:**

An institution-based cross-sectional study was conducted from June 05 to September 30, 2024. The sampling frame consisted of all adult patients admitted to the medical, surgical, and gynecological wards of five public hospitals. A total of 621 participants were selected using simple random sampling from the daily admission logs. Data were collected via a pretested, interviewer-administered questionnaire. EpiData 4.6 and SPSS version 25 were used for data entry and analysis respectively. Logistic regressions were applied to identify factors associated with patient trust. Associations were considered significant at p value < 0.05.

Trust was measured using a validated scale, where “high trust” was operationally defined as a score $\ge$ 32 (based on the mean score of the study population). Bivariable and multivariable logistic regressions were used to identify factors associated with trust, with significance set at $p < 0.05$.

**Results:**

The study achieved a 96% response rate (n = 621). Overall, 47.3% of patients reported high trust in HCPs (95% CI: 43.4–51.4). Factors significantly associated with higher trust included: rural residence (AOR = 2.44; 95% CI: 1.27–4.69), absence of comorbidity (AOR = 1.75; 95% CI: 1.15–2.65), not having health insurance (AOR = 1.53; 95% CI: 1.09–2.14), and a hospital stay of less than 29 days (AOR = 1.89; 1.12–3.20).

**Conclusion:**

Fewer than half of hospitalized adults expressed high trust in HCPs. To bridge this gap, hospital management should implement mandatory patient-centered communication training for clinicians and establish dedicated support protocols for patients with chronic comorbidities. Furthermore, improving the transparency of insurance billing and discharge processes is essential for urban and long-term patients.

## Introduction

Patients’ trust in healthcare providers **(HCPs)** refers to the willingness of patients to accept vulnerability with the expectation that HCPs and the health system will act in their best interest [1,2]. Trust is fundamental to effective healthcare delivery (HCD), influencing health-seeking behavior, treatment adherence, continuity of care, overall health outcomes, and patient engagement in preventive services and public health interventions [3–9]. Globally, declining trust in healthcare systems (HCS) has been reported, driven by factors such as poor patient experiences, communication gaps, and systemic inefficiencies [10–13]. Structural changes in HCD, including increased specialization, technological dependence, and evolving financing models, have further contributed to weakening patient–provider relationships [14–16]. Additional factors such as high healthcare costs, unnecessary investigations, resource limitations, and negative media coverage have also been shown to undermine public trust in HCPs [17–20].

In Sub-Saharan Africa (SSA), including Ethiopia, health systems face persistent challenges such as resource constraints, high patient load, and variability in service quality, which may influence patient trust in healthcare providers. In Ethiopia, issues such as long waiting times, perceived inequities in care, and communication gaps may further affect patient confidence in the HCS, although empirical evidence remains limited. Low levels of trust can discourage timely healthcare utilization, reduce adherence to medical advice, and worsen health outcomes, while also straining patient–provider relationships and increasing stress among healthcare professionals [5–7,9,21–25].

Despite its importance, there is a scarcity of comprehensive and context-specific evidence on patient trust in Ethiopia, particularly at sub-regional levels. Specifically, no studies have examined the level of patient trust and its associated factors in public hospitals of Awi Zone, Northwest Ethiopia. Given the local health system context and limited evidence, assessing patient trust in this setting is essential to inform strategies aimed at improving healthcare quality and patient provider relationships. Therefore, this study aims to assess the level of patient trust and its associated factors among hospitalized adult patients in Awi Zone public hospitals.

## Methods and materials

### Study design, setting and period

We conducted a facility-based study using a cross-sectional design in Awi zone from June 05 to September 30, 2024. Awi zone is one of eleventh Zone in Amhara regional state located in the Northwest, Ethiopia. Administratively, the zone has 10 districts and 6 towns’ administration. It gives the health service for about 1, 381,120 people by its forty-nine health centers and six hospitals one private and five government hospitals. Each Hospital serves for an estimated 150, 000–1,500,000 people. The health service coverage in Awi zone is 74% based on Awi zone health department annual report.

### Population and eligibility criteria

All adult patients who were admitted and taking service in Awi Zone public Hospitals during the study period were included in this study. Whereas patients who were severely ill with coma state were excluded from participating in this study. Hospitalized patients were specifically selected because inpatients typically represent a population with higher acuity and more frequent provider-patient interactions, providing a more robust environment to assess service delivery/evidence-based practices. Furthermore, focusing on inpatients ensured a more homogenous study population, reducing the potential confounding variables associated with the more transient nature of outpatient care.

### Variables

The outcome variable was patients’ trust in HCPs (High trust/Low trust). Whereas the independent variables were socio-demographic factors such as sex, age, residence, and education level. Clinical characteristics such as presence co-morbid disease, frequency of hospital visit and admission, duration of the disease after diagnosis, length of stay in hospital, impaired mobility. Personal related factors such as presence of social support, history of substance use, self-rated health status, social media exposure.

### Operational Definition and Terminologies

Patient trust: The extent to which patients believe that HCPs act in their best interest and will not harm them [1]. High trust: Participants who scored a mean value of ≥32 on the trust assessment scale, based on the scoring approach of the validated Healthcare Relationship. Low trust: Participants who scored a mean value of <32 on the same scale. To assess patients’ level of trust in healthcare providers (HCPs), the Revised HCR Trust Scale–Revised) was employed [26]. Comorbidity: The co-occurrence of two or more medical conditions in the same individual [27]. Short-term stay: A hospital stay lasting less than 29 days, from admission to discharge [28]. Long-term stay: A hospital stay lasting 29 days or more, from admission to discharge [29].

### Sample Size Determination

The sample size was calculated using a single population proportion formula with a 95% confidence level (α = 0.05) and a margin of error of 4% (d = 0.04). and the prevalence of trust 38% from a previous study at Jimma University, Ethiopia [26].

n = (Zα / 2)^2^xp (1 − p)/d^2^ = [(1.96)^2^ × 0.38(1–0.38)]/ (0.04)2 = 565, by adding 10% of non-response rate, the final sample size was 621. A 10% non-response rate was added to the calculated sample size to compensate for potential refusals and incomplete responses, which are commonly reported in hospital-based studies in Ethiopia.

### Sampling Techniques, and Procedures

All five public hospitals in the study area were included: one general hospital and four primary hospitals. The average number of adult admissions over the previous six months was obtained from hospital records and used to estimate the expected number of admissions over a three-month period. The total sample size was then allocated proportionally to each hospital based on its admission volume.

Within each hospital, a sampling frame was prepared using the medical registration numbers of admitted patients. Study participants were selected using a simple random sampling technique after determining the first case by lottery method, and recruitment continued until the allocated sample size was reached.

**Fig 1:**
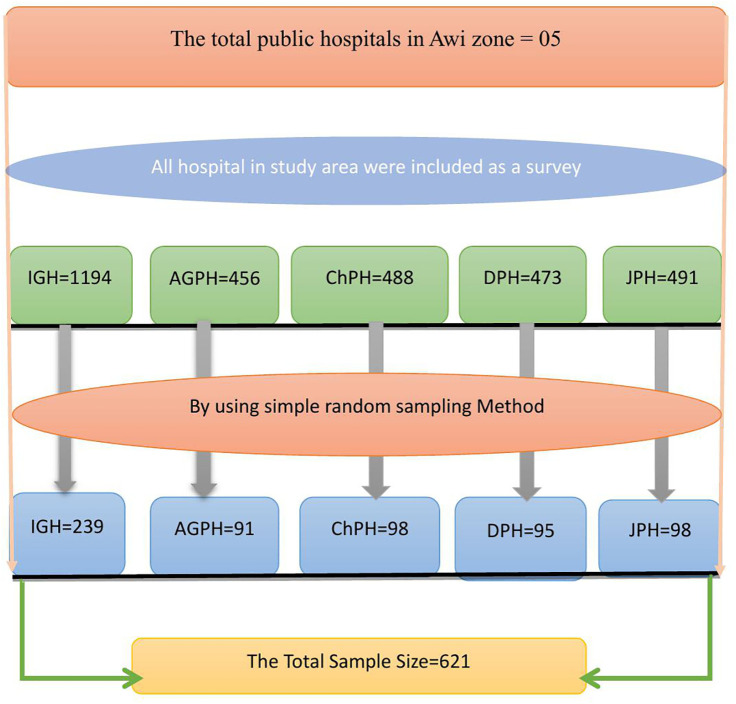
Schematic presentation of the sampling procedure for patients’ trust in healthcare providers and its associated factors among hospitalized adult patients in Awi Zone public hospitals, Northwest Ethiopia, 2024 (n = 621).

To avoid duplicate enrollment during the data collection period, patient records were cross-checked prior to recruitment. In addition, participants were given non-identifiable color-coded cards solely for tracking purposes; these cards contained no personal information and were not accessible to healthcare providers, ensuring confidentiality and minimizing any potential influence on care (Fig 1).

### Data collection tools and procedures

Data were collected using a semi-structured, interviewer-administered questionnaire adapted from previously validated instruments in the literature. The questionnaire comprised five major sections: socio-demographic characteristics, clinical characteristics, individual patient-related factors, organizational factors, and patient–provider trust. These sections were developed based on established frameworks and prior studies [26,30–34]. To assess patients’ level of trust in (HCPs), the Revised HCR Trust Scale–Revised) was employed [35,36]. Trust was measured using a validated scale, where “high trust” was operationally defined as a score $\ge$ 32 (based on the mean score of the study population). Although no formal psychometric validation study of this scale has been conducted in the Ethiopian context, a rigorous cultural adaptation process was undertaken to ensure content validity. This process included forward and backward translation by linguistic and healthcare experts, followed by a pretest involving 5% of the sample (n = 31) to evaluate clarity and relevance. Based on the pretest findings, minor linguistic modifications were made to ensure conceptual equivalence of the construct of “trust” in the local context. Internal consistency of the scale was assessed using Cronbach’s alpha (α = 0.XX), indicating acceptable reliability. The Revised HCR Trust Scale consists of 13 items across six domains: knowledge sharing, emotional connection, professional competence, respect, honesty, and partnership. Each item is rated on a five-point Likert scale ranging from 0 (none of the time) to 4 (all of the time), yielding a total score between 0 and 52. A mean score above 32 indicates a high level of trust in healthcare providers, whereas a score below 32 reflects low trust.

The questionnaire and clinical checklist were initially developed in English, translated into Amharic, and then back-translated into English to ensure linguistic consistency. However, data collection was conducted using the Amharic version. Both primary and secondary data sources were utilized. Primary data were collected through face-to-face interviews with admitted adult patients, while secondary data were extracted from patient medical records. Informed consent was obtained from all participants prior to data collection. Medical charts were selected using a lottery method for the first case, followed by random sampling of subsequent charts. Trained data collectors were responsible for conducting interviews and extracting relevant clinical information from medical records.

### Data processing and analysis

Data were entered into Epi Data version 4.6 and exported to SPSS version 25 for analysis. Descriptive statistics were used to summarize the data. Categorical variables were presented as frequencies and percentages, while continuous variables were summarized using means and standard deviations or medians and interquartile ranges, depending on data distribution.

Both bivariable and multivariable binary logistic regression analyses were performed to identify factors associated with patients’ trust in HCPs. Variables with a p-value of <0.20 in the bivariable analysis were included in the multivariable model to minimize the exclusion of potentially important predictors and to control for confounding effects at an early stage. A p-value of less than 0.05 in the multivariable analysis was considered statistically significant.

Multicollinearity among independent variables was assessed using the variance inflation factor (VIF) and tolerance values. Variables with VIF values greater than 10 or tolerance values less than 0.1 were considered indicative of multicollinearity and were addressed accordingly.

The assumptions of the logistic regression model, including linearity of the logit for continuous variables, were assessed. Furthermore, the Hosmer–Lemeshow goodness-of-fit test was used to assess model fit. The result was not statistically significant (p = 0.74), indicating that the model was considered to have a good fit and was deemed adequate.

### Data quality assurance

To ensure the quality of the data, a two-day training session was conducted for all data collectors and supervisors. The training covered the objectives and relevance of the study, proper use of the data collection tools, and the procedures for data collection. Prior to the actual data collection, a pre-test was conducted on 5% (n = 31) of the total sample size among randomly selected hospitalized adult patients at Finoteselam General Hospital, a facility with similar socio-demographic characteristics to the study sites. The purpose of the pre-test was to assess the clarity, comprehensibility, logical flow, and construction of the questionnaire. Based on the pre-test results, items found to be unclear or confusing were revised accordingly. The reliability of the instrument, as assessed by Cronbach’s alpha, was found to be 0.83, indicating good internal consistency.

The principal investigator and trained supervisors oversaw all data collection activities to maintain consistency and quality. The completed questionnaires were collected daily by the principal investigator. Data were collected by five trained BSc-level nurses and supervised by five MSc-level health professionals; one assigned to each health facility. Each day, the collected data were reviewed for completeness and submitted to the principal investigator. To ensure confidentiality, all data were securely stored in a locked cabinet.

### Ethical consideration

The study was reviewed and approved by the institutional review board (IRB) of Injibara university, college of medicine and health sciences (Ref. No: INU/CMIS/781/17). In addition, official permission letters were obtained from the chief executive officers of the selected hospitals. Prior to data collection, the purpose of the study was clearly explained to each participant by the data collectors. Written informed consent was then obtained from all participants. To ensure confidentiality, participants’ names and other identifying personal information were not recorded or disclosed in any part of the study report.

## Result

### Sociodemographic characteristics of participants

A total of 621 individuals participated in the study, yielding a 96% response rate. The majority of participants were aged 18–45 years (64.9%) and male (60.1%). Most respondents were rural residents (89.4%) and farmers (67.1%). Over half had no formal education (53.0%), and the majority were married (77.8%). Nearly half of the participants (49.8%) were covered by community-based health insurance, and most lived with two or more persons (76.5%) (Table 1).

**Table 1:** Sociodemographic characteristics respondents in Awi zone public hospitals Northwest, Ethiopia, 2024 (n = 621).

S. N	Variables	Category	Frequency	Percent
1	Age	18-45	403	64.9
		≥46	218	35.1
2	Sex	Male	373	60.1
		Female	248	39.9
3	Residency	Urban	66	10.6
		Rural	555	89.4
4	Occupation	Employee	57	9.2
		Merchant	98	15.8
		Farmer	417	67.1
		Others	49	7.9
5	Education	No formal education	329	53
		Formal education	292	47
6	Marital status	Single	138	22.2
		Married	483	77.8
8	Community health insurance (CHI)	Yes	309	49.8
		No	312	50.2
9	Living arrangement	Live alone	28	4.5
		Live with one person	118	19
		Live with Two and above persons	475	76.5

### Clinical characteristics of the respondents

Of the total participants, 173 (27.9%) had one or more comorbid conditions. The majority of respondents, 523 (84.2%), had a disease duration of ≤179 days, whereas 42 (6.8%) had a duration of 180–365 days and 56 (9.0%) had a duration of ≥366 days. Most participants, 533 (85.8%), had a hospital stay of less than 29 days, while 88 (14.2%) stayed for 29 days or more. Nearly half of the respondents (295, 47.5%) had impaired mobility. A large proportion of participants, 452 (72.8%), had a history of previous hospital visits, and 358 (57.6%) had been hospitalized at least once in the past year (Table 2).

**Table 2:** Clinical characteristics of the respondents in Awi zone public Hospitals Northwest Ethiopia, 2024(n = 621).

S. No	Variables	Category	Frequency	Percent
1	Co morbid disease	Yes	173	27.9
		No	448	72.1
2	Duration of the disease after diagnosis in days	≤ 179	523	84.2
		180-365	42	6.8
		≥ 366	56	9
3	Hospital stays in days	<29	533	85.8
		≥29	88	14.2
4	Impaired mobility	Yes	295	47.5
		No	326	52.5
5	History of previous Hospital visit	Yes	452	72.8
		No	169	27.2
6	History of previous Hospitalization	Yes	358	57.6
		No	263	42.4

### Personal and Organizational characteristics of respondents

In this study, 394 (63.4%) participants reported good self-rated health status. Similarly, 394 (63.4%) participants had a support person or group during hospitalization. Regarding awareness, 197 (31.7%) of respondents were aware of the current healthcare delivery system, whereas 424 (68.3%) were not. Only 161 (25.9%) participants were knowledgeable about the roles and responsibilities of physicians and nurses.

Additionally, 128 (20.6%) respondents reported having a family member or relative who is a health professional. Access to health information was reported by 197 (31.7%) participants. Among sources of health information, 121 (19.5%) reported health professionals, 65 (10.5%) mentioned television or radio. Regarding facility-related factors, 428 (68.9%) respondents reported availability of medical equipment and investigations, and the majority (597, 96.1%) indicated that a referral system was in place (Table 3).

**Table 3:** Personal and Organizational factors associated with trust in HCPs in Awi Zone, Northwest Ethiopia, 2024 (n = 621).

S.No	Variables	Category	Frequency	Percent
1	Current health status self report	Good	394	63.4
		Poor	227	36.6
2	Presence of support person/group	Yes	394	63.4
		No	227	36.6
3	Aware of current healthcare delivery system	Yes	197	31.7
		No	424	68.3
4	Knowning of duty and responsibility physician and Nurses	Yes	161	25.9
		No	460	74.1
5	Family member/relative who are health profession	Yes	128	20.6
		No	493	79.4
6	Access of Health information	Yes	197	31.7
		No	424	68.3
7	Source of Health information	Health profession	121	19.5
		Tv and radio	65	10.5
		Other social media	11	1.8
8	Current substance use	Yes	10	1.6
		No	611	98.4
11	Types of Current Substance use	Alcohol	4	.6
		Chat	4	.6
		Cigaritee	2	.3
	Availablity of medical equipment and investigation	Yes	428	68.9
		No	193	31.1
12	Referal system	Yes	597	96.1
		No	24	3.9

### Prevalence of patients’ trust on HCPs in Awi Zone. Northwest Ethiopia

**Fig 2:**
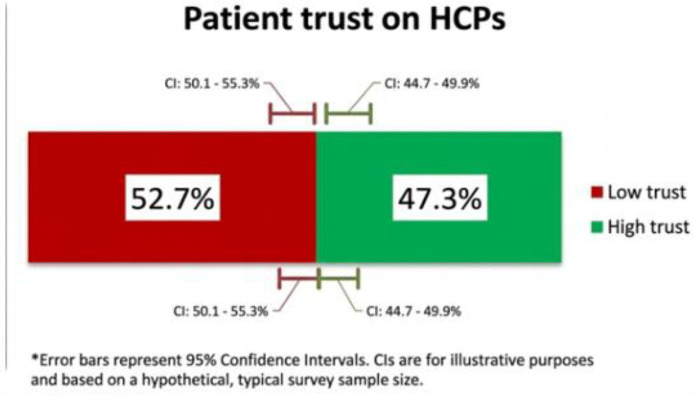
The levels of Patients’ trust in healthcare providers and its associated factors among hospitalized adult patients in Awi Zone public hospitals, Northwest Ethiopia, 2024 (n = 621).

In the current study,the mean (± SD) trust score of respondents toward healthcare providers was 32 ± 15.2, with a minimum score of 4 and a maximum score of 52. Overall, the prevalence of patients’ trust in HCPs was 47.3% (95% CI: 43.4–51.4) (Fig 2).

### Factors associated with patients trust in HCPs

In the multivariable logistic regression analysis, four variables were significantly associated with patients’ trust in healthcare providers. Patients residing in rural areas were more than twice as likely to have high trust compared to urban residents (AOR = 2.44, 95% CI: 1.27–4.69; p = 0.007). Respondents without comorbid conditions were also more likely to report higher trust than those with comorbidities (AOR = 1.75, 95% CI: 1.15–2.65; p = 0.009). Participants without community-based health insurance had higher odds of trust compared to insured individuals (AOR = 1.53, 95% CI: 1.09–2.14; p = 0.010). Additionally, patients with a shorter hospital stay (<29 days) were more likely to have higher trust than those with longer stays (AOR = 1.89, 95% CI: 1.12–3.20; p = 0.018) (Table 4).

**Table 4:** Logistic regression analysis for factors associated withpatient trust in HCPs in Awi Zone, Northwest Ethiopia, 2024, (n = 621).

S.N	Variables	Category	Pts trust in HCPs		COR	AOR (CI)	P -Value
			Low trust	High trust			
1	*Residency*	Rural	280 (50.5)	275 (49.5)	3.78 (2.05-6.98)	2.44 (1.27-4.69)	**0.007** ^**^
		Urban	14 (21.2)	52 (78.8)	1		
2	Comorbidity	No	233 (52)	215 (48)	1.99(1.38-2.86)	1.75 (1.15-2.65)	**0.009** ^**^
		Yes	61(35.3)	112 (64.7)	1		
3	Length of Stay in hospital in days	<29	268(50.3)	265 (49.7)	2.41(1.48-3.93)	1.89 (1.12-3.20)	**0.018** ^**^
		≥29	26(29.5)	62 (70.5)	1		
4	Self reported current health Status	Good	258(49.8)	260 (50.2)	1.85(1.19-2.87)	1.28(0.79-2.08)	0.311
		Poor	36(25.2)	67 (74.8)	1		
5	Hospitalization history	Yes	192(53.6)	166 (46.4)	1.83(1.32-2.52)	1.37 (0.96-1.96)	0.087
		No	102(38.8)	161(61.2)	1		
6	Awarenes of the current H.C delivery system	Poor	221(52.1)	203 (48.9)	1.87 (1.31 −2.61)	1.31 (.88-1.96)	0.180
		Good	73 (37.1)	124 (62.9)	1		
7	Presence of CHI	No	172 (55)	140 (45)	1.88 (1.37-2.59)	1.53 (1.09-2.14)	**0.010** ^**^
		Yes	122 (39.5)	187 (60.5)	1		
8	Previous hospital visit before current admission	Yes	226 (50)	226 (50)	1.49(1.04-2.13)	1.26(0.84-1.89)	0.31
		No	68(40.2)	101(59.8)	1		

## Discussion

This study found that 47.3% (95% CI: 43.4–51.4) of participants reported high trust in HCPs. This finding is comparable to a study conducted in Saudi Arabia (47.8%) [37], but higher than reports from Jimma Medical Center, Ethiopia (38%) [26], and Nigeria (28.1%) [38]. It also exceeds findings from China (24.3%) [39], and the United States (30–32%) [33,40]. These differences may reflect variations in study populations, measurement tools, and data collection methods. For example, the present study employed a validated multi-item scale and face-to-face interviews, which may have facilitated more engaged responses compared to telephone-based surveys used in some high-income settings [17,40–42]. In study in Nigeria, only 5% of respondents are enrolled in national health insurance, and 70% lack community-based health insurance (CHI) In contrast, 93% of the participants in the present study were enrolled in such schemes, which likely fostered greater trust in the healthcare system [42–44].

However, the observed level of trust is lower than that reported in Croatia (65.6%) and studies from China (64.2%–83.4%) [13,31,45,46]. This difference may be related to broader health system factors. In Ethiopia, constraints such as limited healthcare resources, variability in service quality, and gaps in patient-centered care may influence patients’ perceptions and trust. These findings suggest that strengthening service quality, communication, and system responsiveness may be important for improving patient trust.

Residence was significantly associated with trust, with rural participants more likely to report higher trust compared to urban residents. Similar patterns have been reported in China and Nigeria [31,47]. In the Ethiopian context, this difference may reflect variations in expectations, prior experiences with healthcare services, and perceived alternatives. Rural patients may have fewer healthcare options and different benchmarks for evaluating care, which could influence reported trust levels. However, this finding should be interpreted cautiously, as the study did not directly assess mechanisms underlying this association.

Furthermore, participants without comorbid conditions were more likely to report higher trust than those with comorbidities. This may be related to differences in care experiences, as patients with multiple conditions often require more frequent interactions with the health system and more complex care. If these interactions do not meet expectations in terms of communication, coordination, or continuity, trust may be affected. However, given the cross-sectional nature of the study, no causal relationship can be established. Interestingly, individuals without com CHI were more likely to report higher trust compared to insured participants. While this finding is consistent with finding from China [31], its interpretation in the Ethiopian context requires caution. One possible explanation is that insured patients may have more frequent contact with the healthcare system and thus more opportunities to perceive service limitations, such as delays, administrative procedures, or perceived differences in service delivery. Alternatively, differences in expectations between insured and uninsured patients may influence reported trust. However, this study did not explore these mechanisms directly, and further context-specific research is needed to better understand this relationship.

Similarly, shorter hospital stays were associated with higher reported trust. This may reflect differences in patient experiences during hospitalization, such as perceived efficiency of care, communication, or clinical improvement. However, it is equally plausible that patients with more severe conditions require longer stays and may have more complex or less satisfactory experiences. Therefore, this association should not be interpreted as causal.

### Strengthen of Study

This study presented several methodological strengths that enhance the credibility and generalizability of its findings. First, the use of both primary and secondary data collection methods enabled the integration of rich, context-specific information with relevant background data, thereby offering a more comprehensive and nuanced understanding of patients’ trust in HCPs. Second, the assessment of trust was conducted using the Revised Health Care Relationship Trust Scale (HCR Trust Scale–Revised), a validated and standardized tool. This enhanced the reliability and validity of the measured outcomes, ensuring that the construct of trust was captured accurately. Finally, the inclusion of participants from all five public hospitals in the Awi zone minimized selection bias and reduced design effect, thereby strengthening the representativeness and generalizability of the study results across similar settings.

### Limitation of this study

The study has several limitations. Although the trust measurement scale was translated and its internal consistency was assessed, exploratory factor analysis was not conducted to evaluate its construct validity in the Ethiopian context. Therefore, the dimensional structure of the scale may not be fully established, which could affect the interpretation of the findings. The cross-sectional design of the study limits the ability to establish causal relationships between the variables, as data were collected at a single point in time. In addition, the use of interviewer-administered questionnaires may have introduced social desirability bias, as respondents might have provided answers they perceived as more acceptable rather than their true opinions.

The use of self-reported data may also be subject to recall bias, which could affect the accuracy of the responses. Furthermore, the study was conducted in a single zone in Ethiopia, which may limit the generalizability of the findings due to the country’s cultural, linguistic, and social diversity. The inclusion of only hospitalized patients may introduce sampling bias, as their experiences and levels of trust may differ from those of outpatients, thereby limiting representativeness. Finally, future studies are recommended to further validate the instrument using factor analysis and to address these limitations in broader and more diverse populations.

## Conclusion and recommendations

This study identified key factors associated with higher levels of trust in HCPs among hospitalized patients. Specifically, rural residence, shorter hospital stays (<29 days), absence of comorbid conditions, and lack of CHI were significantly associated with greater patient trust in HCPs. These findings highlight important areas where HCS and providers can implement targeted, actionable strategies to foster and sustain patient trust. Hospitals and health facility administrators should strengthen patient–provider communication by developing tailored communication strategies, particularly for urban patients and those with longer hospital stays or comorbid conditions. This may include improving clarity of information, increasing patient engagement during consultations, and ensuring responsiveness to patient concerns.

In addition, healthcare facilities should enhance patient-centered care by improving service delivery, reducing waiting times, and ensuring continuity of care, especially in urban settings where lower levels of trust were observed. Health professionals should also emphasize transparency, empathy, and responsiveness in their interactions with patients, particularly those with comorbidities or prolonged hospitalization. Policymakers should review and strengthen CHI programs by addressing potential trust-related barriers. This includes improving transparency in enrollment and benefit packages, enhancing service quality, and increasing public awareness of the advantages of such schemes, particularly in urban areas where trust was lower.

Furthermore, targeted health literacy interventions should be implemented to improve patients’ understanding of healthcare services and insurance systems, thereby supporting informed decision-making and strengthening trust. Efforts should also focus on ensuring equitable healthcare access and improving service quality across both rural and urban settings to reduce disparities in patient trust. Finally, future research should include qualitative and mixed-methods studies to explore in greater depth the contextual and interpersonal factors influencing patient trust. Such studies can provide more nuanced insights to inform the design of effective, context-specific interventions to strengthen trust in healthcare systems.

## Supporting information

S1 File SPSS data. (SAV)

## Abbreviation:

CHI: Community Health Insurance
HCPs: Health Care providers
HCR: Health Care Relation
HCD: Health care delivery
HCS: Health Care System
SSA: Sub Saharan Africa
SPSS: Statistical Package for social science
VIF: variance inflation factor
